# Breast cancer and the pill.

**DOI:** 10.1038/bjc.1989.174

**Published:** 1989-05

**Authors:** R. D. Farmer


 © The Macmillan Press Ltd., 1989

### LETTER TO THE EDITOR

### Breast cancer and the pill

**Sir** – In their recent paper Kay & Hannaford (1988) tentatively concluded that oral contraceptive use 'before the first term birth may be associated with an increased rate of presentation of breast cancer in women under the age of 35 years'. They point out that studies designed to explore the association between breast cancer and oral contraceptive use have produced inconsistent results and are rightly cautious in their overall interpretation of their study findings. When different studies of the same subject produce conflicting results, it is essential to evaluate each study critically in order to ascertain how the conflicts might have arisen. Kay and Hannaford's study has several problems which may invalidate even the most tentative of conclusions.

The study is based on a cohort recruited in 1968–69 of about 23,000 women, who were then current users of oral contraceptives, and a similar number of women who were not using oral contraceptives. The patients were recruited through a network of research orientated general practitioners. In an earlier paper (Royal College of General Practitioners, 1974) the advantages of involving the general practitioners in this way were explained; it was said that it would enable the investigators to obtain complete data and that it would facilitate follow-up. In view of the latter claim it is disappointing that by March 1985 (17 years), only 18,000 (38.3%) of the original 47,000 women were still under observation. This represents an average loss to follow-up of 3.6% per year. Moreover, from their Table I the study so far has yielded 406,836 woman years of experience – this is an average of 8.66 years per woman or just over 50% of what was theoretically possible. No information is given about the women lost to follow-up nor is there any explanation of the loss. It is possible that the women lost to follow-up were so different from those who were not as to invalidate the analyses.

The breast cancer rates quoted were standardised for 'age and parity at the time of diagnosis and daily cigarette consumption and social class at recruitment'. Presumably this procedure was adopted in order to eliminate the effects of important confounding variables. The authors accept that age of mother at first birth is an important determinant of breast cancer risk but they did not foresee the need to collect this piece of information when the study was designed. Their justification for adopting parity as a proxy for age at first pregnancy is that Macmahon *et al.* (1970) showed that parity was correlated with age at first pregnancy. Macmahon *et al.*'s work was undertaken in the 1960s, mostly before the patients were recruited to the Kay & Hannaford study, and was concerned with residents of North America. It is unlikely that the correlation between age of first pregnancy and parity that they noted has been sustained following the introduction of modern contraceptives and even more unlikely that the patterns of family building observed in the USA in the 1950s and 1960s could be applied to UK women in the 1970s. The authors' contention that 'the effects of any differences in the age at first birth between groups should be reduced by standardisation for parity' cannot be sustained on the information that is offered.

From a number of case–control studies there is evidence that breast cancer risk is associated with smoking. It is therefore reasonable to attempt to adjust the rates for variations in smoking habits when exploring the relationship between oral contraceptive use and breast cancer. However, it is questionable whether the daily consumption of cigarettes at recruitment to the cohort should form the adjustment. In the intervening 17 years it is possible that the smoking habits of the women studied varied considerably, particularly as the smoking habits of women may well change because of pregnancy. There is no *a priori* reason to suppose that the interval between exposure to tobacco and the occurrence of breast cancer is such that this single historical measure should be appropriate.

The standardisation for social class measured at the time of recruitment to the cohort presents similar problems to those of cigarette smoking. It has been established that there is an association between carcinoma of the breast and social class at the time of diagnosis. It is unlikely that the social class distribution of a group of women will be sufficiently stable over 17 years to justify using the social class at recruitment to the study as a proxy for social class at diagnosis.

The use of exposed and non-exposed woman years as the main denominator in many of the analyses makes it difficult to establish how many different women were in the subcategories described, but it is possible to highlight an important anomaly. Table III of Kay & Hannaford's paper sets out breast cancer risk by parity in ever users and controls, and the risk ratios (after standardisation for age at diagnosis and social class and smoking habit at recruitment). The risk ratio is computed by dividing the incidence rates among the ever users by the incidence rates among the never users (controls). This indicates a significant increase in risk of between 2.2 and 17.1 among the ever exposed of parity 1 compared to controls of the same parity. Using the same methodology it is clear that parity has different effects on controls compared to the ever users (see Table).

| | *Ever users* | | *Controls* | |
|---|---|---|---|---|
| *Parity group* | *Standardised rate (TWY)* | *Risk ratio (para 0=1)* | *Standardised rate (TWY)* | *Risk ratio (para 0=1)* |
| 0 | 0.42 | 1.00 | 0.37 | 1.00 |
| 1 | 0.93 | 2.21 | 0.16 | 0.43 |
| 2+3 | 0.65 | 1.55 | 0.65 | 1.76 |
| 4+ | 0.48 | 1.14 | 0.68 | 1.84 |

Earlier in their paper the authors advanced the view that parity is correlated with age at first birth and that parity could therefore be used as a proxy for that important variable. Their data show that the risk among both groups of nulliparous women is lower than among parous women and that the apparent slope of the risk ratio according to parity is paradoxical between the ever users and never users. This observation must bring into question their findings set out in Table IV, which show an apparent increase in risk among para 1 women exposed to oral contraceptives for between 2 and 7 years and for women aged 30–34 years.

Yours etc.,

R.D.T. Farmer,
Dept. of Community Medicine,
Charing Cross and Westminster Medical School,
17 Horseferry Road,
London SW1P 2AF, UK.

### References

KAY, C.R. & HANNAFORD, P.C. (1988). Breast cancer and the pill – a further report from the Royal College of General Practitioners' oral contraception study. *Br. J. Cancer*, **58**, 675.

MACMAHON, B., COLE, P., LIN, T.M. and 6 others (1970). Age at first birth and breast cancer risk. *Bull. WHO*, **43**, 209.


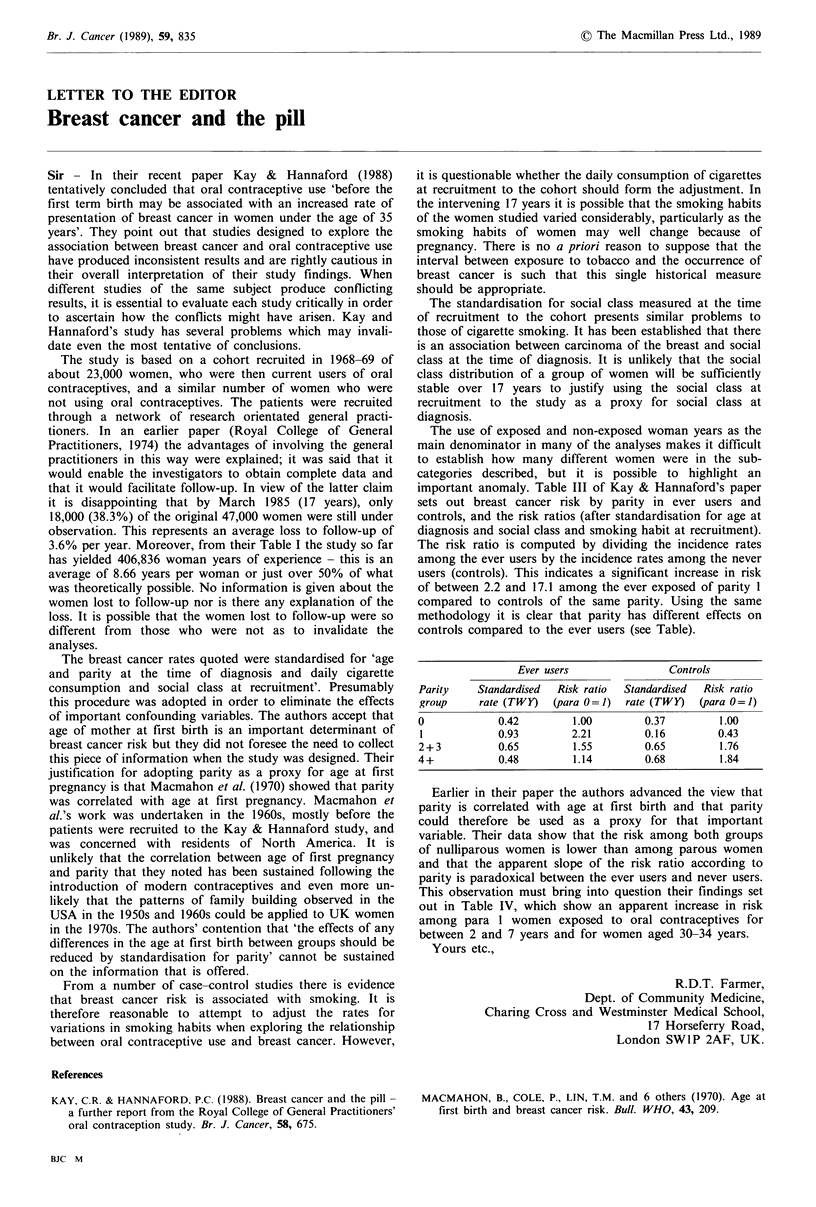


## References

[OCR_00142] Kay C. R., Hannaford P. C. (1988). Breast cancer and the pill--a further report from the Royal College of General Practitioners' oral contraception study.. Br J Cancer.

[OCR_00149] MacMahon B., Cole P., Lin T. M., Lowe C. R., Mirra A. P., Ravnihar B., Salber E. J., Valaoras V. G., Yuasa S. (1970). Age at first birth and breast cancer risk.. Bull World Health Organ.

